# *Vibrio* Pathogens: A Public Health Concern in Rural Water Resources in Sub-Saharan Africa

**DOI:** 10.3390/ijerph14101188

**Published:** 2017-10-07

**Authors:** Charles A. Osunla, Anthony I. Okoh

**Affiliations:** 1 SAMRC Microbial Water Quality Monitoring Centre, University of Fort Hare, Alice, Private Bag X1314, Alice 5700, South Africa; aokoh@ufh.ac.za; 2 Applied and Environmental Microbiology Research Group (AEMREG), Department of Biochemistry and Microbiology, University of Fort Hare, Alice 5700, South Africa; 3 Department of Microbiology, Adekunle Ajasin University, P. M. B, Akungba-Akoko 34211, Ondo-State, Nigeria

**Keywords:** *Vibrio* pathogens, rural water resources, public health, sub-Saharan Africa

## Abstract

Members of the *Vibrio* genus are autochthonous inhabitants of aquatic environments and play vital roles in sustaining the aquatic milieu. The genus comprises about 100 species, which are mostly of marine or freshwater origin, and their classification is frequently updated due to the continuous discovery of novel species. The main route of transmission of *Vibrio* pathogens to man is through drinking of contaminated water and consumption inadequately cooked aquatic food products. In sub-Saharan Africa and much of the developing world, some rural dwellers use freshwater resources such as rivers for domestic activities, bathing, and cultural and religious purposes. This review describes the impact of inadequately treated sewage effluents on the receiving freshwater resources and the associated risk to the rural dwellers that depends on the water. *Vibrio* infections remain a threat to public health. In the last decade, *Vibrio* disease outbreaks have created alertness on the personal, economic, and public health uncertainties associated with the impact of contaminated water in the aquatic environment of sub-Saharan Africa. In this review, we carried out an overview of *Vibrio* pathogens in rural water resources in Sub-Saharan Africa and the implication of *Vibrio* pathogens on public health. Continuous monitoring of *Vibrio* pathogens among environmental freshwater and treated effluents is expected to help reduce the risk associated with the early detection of sources of infection, and also aid our understanding of the natural ecology and evolution of *Vibrio* pathogens.

## 1. Introduction

Freshwater bodies serve as the main water resources in rural areas used for drinking, cooking, and irrigation for agriculture in most communities that have little or no access to potable, safe water. They easily become polluted as a result of fast population growth, land development along river banks, and urbanization [1]. Continuous pollution has resulted in various water-associated disease epidemics in both developed and developing countries [2,3]. More than 50% of the 663 million people worldwide who lack access to safe water reside in Sub-Saharan Africa, predominantly in rural areas [4]. This leads to poor health due to various water-related illnesses. However, access alone is not enough to guarantee better health. Insufficient hygienic practices can also lead to the contamination of safe water after it leaves the water point, making it unsafe to drink. Globally, 80% of wastewater flows back into the ecosystem without being treated or reused, contributing to a situation where around 1.8 billion people use a source of drinking water contaminated with faeces, putting them at risk of contracting cholera, dysentery, typhoid, and polio [5]. Mainly in low-income areas of cities and towns within developing countries, a large proportion of wastewater is discharged directly into the closest surface water drain or informal drainage channel, sometimes with very little or no treatment at all [5]. In addition to household effluent and human waste, urban-based hospitals and industries such as small-scale mining and motor garages often dump highly toxic chemicals and medical waste into the wastewater system. Even in developed countries where wastewater is collected and treated, several reports have established that most wastewater treatment plants (WWTPs) do not completely remove contaminants from sewage waters based on the system used [6], thus releasing effluents with varying matrices of contaminants at a specific point source into water bodies such as rivers, streams, and lakes. Despite recent advances in water quality and wastewater treatments, waterborne diseases still pose a major threat to public health worldwide [7]. As these receiving water bodies are the only available sources of potable water, their contamination has resulted in many waterborne diseases such as diarrhoea, gastroenteritis, and cholera in children, adults, and refugees in various developing countries such as Nigeria, Rwanda, Congo, Zimbabwe, Sudan, Afghanistan, Chile, and Brazil [8]. In rural areas of sub-Saharan countries like South Africa, rivers play an essential role in the life of the people for social, cultural, and religious purposes. The general public at large is directly affected by the prevailing poor quality of river water which has required the regulation of the biological quality of both effluents and the receiving waterbodies. The lack of policy and management of drinking water safety issues is an important issue which is believed to be a major factor causing the health and safety problems in developing countries. This calls for concerted efforts tailored towards ensuring that more researches are carried out for addressing the paucity of policies guiding the safety of drinking water in sub-Saharan Africa [9].

Over the years, several researchers have focused on the sternness of diseases caused by *Vibrio cholerae* leaving out relatively minor *Vibrio* species of medical interest, some of which are described as emerging pathogens able to cause mild to severe human diseases. The *Vibrio* species are aboriginal gram-negative bacilli that inhabit freshwater and estuarine water environments with a wide range of salinity and temperature values, where they predominantly persist in a culturable and non-culturable state [10,11]. They are considered to exhibit both fermentative and respiratory metabolisms. Several investigations have also shown the prevalence of *Vibrio* species in surface water throughout the world, and their prevalence in the environment is influenced by season, location, and the analytical methods employed [12]. Dozens of known species have been estimated to establish disease conditions in humans [13,14]. They are usually linked to eruptions of *Vibrio* infections as a result of consuming undercooked seafood and water contaminated with sewage or the exposure of skin wounds to aquatic environments and animals [15,16,17]. The pathogenic *Vibrio* species of health relevance which are generally transmitted through water and seafood include *Vibrio parahaemolyticus*, *V. cholerae*, *Vibrio vulnificus*, *V. tubiashi*, and *Vibrio fluvial* is [18,19]. The *Vibrio* genus comprises about 100 species which are mostly found in marine and surface water, and this is subject to continuous updates as a result of the discovery of new species [20]. *V. vulnifcus*, *V. parahaemolyticus*, and *Vibrio mimicus* are considered to be the toxigenic food-borne pathogens [21,22].

*V. cholerae* is an example of a non-invasive organism, which only affects the small intestine via the release of enterotoxin and is the etiological agent of cholera, whereas *V. parahaemolyticus* and *V. vulnificus* are considered as intrusive microorganisms largely affecting the colon. *V. fluvialis* and *V. vulnificus* are considered as emerging human and foodborne pathogens, respectively, and are linked with outbreaks and sporadic cases of severe diarrhea [23,24,25]. *V. vulnificus* symptoms include blistering gastroenteritis, skin wounds, or a disease condition known as primary septicemia and the infection is very dangerous to people who have long-term chronic liver disease [26]. Halophilic *Vibrio* species are known to cause mild infections in humans, but can also cause high morbidity, mortality, or infections in fish and other aquatic animals [27,28]. Generally, the outbreak of *Vibrio* species in aquaculture has a direct impact on the economy of a country and also serves as a threat to public health. In this review, we carried out an overview of pathogenic *Vibrio* species in rural water resources of Sub-Saharan Africa and its implications on public health.

## 2. *Vibrio* Species

The bacteria genus *Vibrio* is considered to be among the natural dwellers of aquatic environments which play essential roles in maintaining the aquatic ecosystem. Vibrios are characterized as gram-negative organisms, have straight or sometimes curved rod-like shapes, and are around 1.4–2.6 μm in length [29]. They can be motile or non-motile; motile species move about with the aid of three flagella at one end. *Vibrio* species usually produce many horizontal unsheathed flagella. They are chemoorganotrophic which are characterized as non-endospores formers and grow in the absence of molecular oxygen. They are different from pseudomonads in that they undergo fermentative as well as respiratory metabolism and are generally positive to an oxidase test [30]; oxygen is the actual final electron acceptor. They are not capable of fixing nitrogen; the usual source of nitrogen is ammonium salts. Nearly all *Vibrio* pathogens are positive to an oxidase test with the exception of *V. metschnikovii* [29].

Vibro-static agent 0/129 has been reported to have an effect on most *Vibrio* species and this serves as the diagnostic test [31]. They exhibit the unique capability to halt and absorb a broad range of carbon, phosphorus, and nitrogen substrates [32,33,34], as well as the ability to secrete exterior enzymes chitinase and laminarase, which make abundant nutrients available to the indigenous microbes [35,36]. Furthermore, they have developed an adaptive mechanism to the ever changing environmental conditions which includes the changing of size to an ultra-microbial morphology (<0.4 µm diameter) [37]. *Vibrio* species are halophilic in nature, requiring about 2–3% sodium chloride (NaCl) for optimum development [38,39]. *Vibrio* species apart from *V. mimicus* and *V. cholerae* are referred to as halophilic organisms because they do not grow on media that is void of the addition of sodium chloride [40]. The potential significance of salinity to *Vibrio* species growth shows the dynamics of abundance in the aquatic ecosystem [41,42].

The genus *Vibrio* has experienced various modifications in recent years. Many studies concentrate on cholera as a result of the havoc that the disease has inflicted on public health, but recently, ample studies have established some of the minor *Vibrio* species to be of important health concern. This minor species are termed as emerging pathogens capable of causing slight to serious diseases in man [43,44], marine vertebrates, and invertebrates [45,46,47]. The details of selected *Vibrio* pathogens that are of medical relevance are listed in Table 1. Over a hundred species are presently in this genus, twelve of which are regarded as human pathogens [48].

Pathogenic *Vibrio* species of human origin are broadly categorized based on the kinds of disease conditions that they exhibit, including one group causing extra-intestinal illnesses and the other group causing gastrointestinal diseases. *Vibrio* species-specific diagnostic tests have been long-established with the aid of biochemical techniques, which are given in Table 2 [31]. Some *Vibrio* species, which are known to emit light, also exhibit symbiotic relationships with squids and various aquatic organisms [50]. Other *Vibrio* species are known to be morbific to certain organisms which include fish, coral, and frogs [51,52,53].

## 3. Ecology of *Vibrio* Species

Vibrios are autochthonous to the oceanic, estuarine, and freshwater ecosystem [54]. They are found in sediments [55] and are known to produce biofilms on surfaces [56,57]. They either swim freely in the water column, [58] or adhere to/live associated with other organisms [20,59]. Moreover, ample numbers of *Vibrio* species have developed adaptive features that enable them to predominantly thrive in salty and even riverine environments [60]. The study of the ecology of *Vibrio* species has been in existence for a long time, owing to the fact that many species are of medical importance to both human and animals [61]. Naturally occurring Vibrios in aquatic environments are well documented considering their great importance in the mineralization of organic matter and other nutrients [62,63]. Because *Vibrio* species are selectively scraped by aquatic flagellates, it is believed that they facilitate the degradation of organic matter in water milieu [64].

They also have the ability to break down chitin, which has been reported as one of the major sources of amino sugars in aquatic environments [62]. In addition, *Vibrio harveyi* secretes an average of ten unique enzymes capable of degrading chitin [65,66]. Accordingly, this might justify the ubiquitous occurrence of *Vibrio* species in aquatic systems [62]. Previous studies have highlighted several *Vibrio* species that are resident in freshwaters which are being transferred through flood run-off to marine environments [67]. Members of the *Vibrio* genus are not always introduced into the aquatic ecosystem with faecal pollution, unlike enteric pathogens that are found in aquatic environments as a result of the indiscriminate discharge of wastewater. Some hypotheses have attempted to explain the prevalence of some pathogenic *Vibrio* species in coastal areas. Several studies have also proposed terrestrial and aquatic animals as reservoirs for virulent genes in the environment [68]. In particular, bivalves and other filter feeding marine animals have been reported to concentrate ample numbers of bacteria in their tissues [69,70]. During warm periods in temperate waters, almost 100% of oysters harbour *Vibrio* species, and an annual study on the Southwest coast of India shows that 57% of all oysters contained pathogenic Vibrios [71].

Numerous studies that have investigated the distribution of *Vibrio* species suggest that pathogenic subpopulations of the genus *Vibrio* are potential reservoirs for disease epidemics [72,73], mainly in sub-Saharan Africa, where access to potable water is lacking [63,74], and/or in countries where the eating of undercooked oysters is most prevalent [22,75]. Previous studies have established that it is almost impossible to understand the effect of single physicochemical parameters on *Vibrio* species since all parameters are interdependent and the influence of the environmental conditions varies from one species to another [67]. The incidence and the rate distribution of *Vibrio* species have been linked to a vast array of environmental factors, most notably organic matter, salinity, temperature, and the association with aquatic animals depending on the pathogen and its habitat, and the geographic location [76,77,78]. Dissolved oxygen [79,80], chlorophyll [81,82,83], and plankton [84,85,86,87] have also been found to be important in the ecology of the *Vibrio* species. However, the effects of these environmental parameters have been reported to be species dependent [88,89].

Globally, climate change is anticipated to have direct or indirect effects on environmental conditions. The noticeable increase in water temperatures, both in oceans and coastal waters, is dependent on higher atmospheric temperatures [90]. An increased temperature in freshwater tends to increase the densities of *Vibrio* species in aquatic animals and this has been implicated in diarrhea and gastroenteritis outbreaks in countries with previous epidemic cholera and temperature-based models [76,77]. A high atmospheric water temperature is one of the factors that suggest the presence of *Vibrio* species as many studies have documented their abundance in warmer waters above 15 °C [12,91,92]. Slight increases in water temperature have been found to greatly influence the microbial load of *Vibrio* species in the face of climate change [93]. Cases of disease outbreak were reported in Hurricane Katrina in the United States in 2005 as result of pathogens [94]. At certain temperatures above 15 °C, attachment to chitin increases considerably owing to an increase in the appearance of the mannose-sensitive haemagglutinin pilus and the colonization factor, an N-acetylglucosamine binding protein [95,96].

At microenvironment temperatures, irrespective of aerated medium uptake, *Vibrio fluvialis* has been shown to exhibit the innate ability to survive and proliferate in saltwater microcosms for almost two weeks [97]. Studies have shown that *Vibrio fluvialis* in aquatic environments can be viable for up to twelve months and still be capable of establishing an infection, and it was later recovered from sediments from a viable but nonculturable stage, after more than six years [98]. According to a study conducted by Scheldt [99], it was observed that runoff from rivers might affect the salinity level in the receiving water bodies, which in turn enhances the proliferation of *Vibrio* species. Similarly, [100] revealed that the densities of *Vibrio species* in the Chesapeake lagoon have a relationship with the rate at which the river flows. They were able to establish the direct impact of runoff on the salinity as a result of dilution. Moreover, climate change is considered to have a noticeable effect on the volume of nutrients in the water since variations in the introduction of nutrients such as through freshwater runoff, as well as the addition of organic carbon, brings about changes in precipitation forms [101].

*Vibrio* species have been reported to be capable of surviving in many different environmental conditions due to the development of a spectrum of adaptive responses to nutrient deficit, variations in salinity and temperature, and a resistance to predation by heterotrophic protists and bacteriophage. One such approach is to undergo a change into a dormant or viable but non-culturable (VBNC) state during harsh situations [102,103]. The significance of the viable but non-culturable state in cholera epidemiology was revealed by incubating a viable but non-culturable state of *V. cholerae* in freshwater microcosms which actively expressed virulence and colonization traits [104]. Likewise, the formation of a biofilm by *Vibrio* species on the exoskeletons of crustaceans and other marine organisms is a survival strategy during famishment and/or other environmental difficulties [105,106,107]. In biofilms, bacteria are believed to conserve and absorb nutrients, resist antibiotics, and create promising associations with other bacteria or hosts. In a conducive environment that is usually season reliant, they are known to revert to the active vegetative state for development and proliferation [108].

## 4. Pathogenicity of *Vibrio* Species

The clinical manifestation of *Vibrio* infections commences with the drinking of contaminated water or the eating of mishandled marine products [24]. After passing through the acidic wall of the stomach, it attaches itself to the thin tissue lining the small intestine with the aid of toxin-coregulated pili (TCP) [109] and with establishment factors like accessory colonization factor, diverse haemagglutinins, and core-encoded pilus. Human pathogenic species are known to produce several extracellular factors including haemolysin, cytotoxin, siderophore, phospholipase, collagenase, enterotoxin, and haemagglutinin [110,111]. Taking into account all of the virulence properties, haemolysin, enterotoxin, and cytosine have a direct link to the clinical manifestation; conversely, siderophore and haemagglutinin are involved in the establishment of *Vibrio* pathogen disease conditions [28]. One of the important means by which pathogens establish their pathogenicity is through the production of bacterial enzymes. Essential proteolytic enzymes that breakdown the amide bond in proteins and other short amino acids are vital for regulating homeostasis in prokaryotes and eukaryotes. Occasionally, the enzymes produced by virulent *Vibrio* species are found to be toxic to the infected human host [112]. As presented in Table 3, relevant *Vibrio* pathogens associated with human infections produce and form proteolytic enzymes; some of these enzymes are broadly classified as toxic factors processing other protein toxins [49].

Studies have shown that poor sanitation and overcrowding are important factors that promote the persistence of *Vibrio cholerae* in the environment, an etiological agent of cholera [113]. Also, *V. cholerae* is known to express two major virulence factors, namely the cholera toxin (CT), which is borne on filamentous cholera-causing toxin phage (CTX phage); and a colonization factor named toxin co-regulated pilus (TCP), which is one of the crucial intestinal establishment factors and the host receptor for the cholera toxin. The cholera toxin causes prolific squelchy diarrhoea, and the two subsets of the isolates are acquired by lateral gene transfer (LGT) [114]. Toxigenic *V. cholerae* growth phase requires two main stages: One entails the ability to cleave and grow on biotic surfaces in a fairly oligotrophic aquatic environment with low osmolarity; while the other requires the successful colonization of a eutrophic, biochemically challenging human intestine populated by a highly diverse commensal host flora [115]. The release of toxins in a living host leads to the discharge of copious squelchy diarrhea that releases the causative organism back into the environment where it is capable of further infecting additional individuals through the consumption of contaminated water, or having access to the environmental stage of its lifecycle. At the environmental stage, the environment avails *V. cholerae* with a substantial benefit to transform by obtaining different genes from other bacteria via lateral gene transfer (LGT). *Vibrio cholerae* is naturally found in aquatic environments and is believed to coexist with zooplankton. When growing on chitin, which is the basic unit of planktonic crustaceans, the organism initiates a growth pattern known as natural competence which enables *Vibrio cholerae* to absorb novel DNA that aids the virulence of the toxigenic strains, via competence-specific DNA uptake machinery.

Considering all the known *Vibrio* pathogens that have been well documented, the most significant of them are *V. cholerae* subgroups O1 and O139, which have been recognized to cause cholera [116]. Intensive study has been pursued for some of the O1 and O139 serotypes that are known to produce an array of virulence genes mostly of the TCP Pathogenicity Island, tcpA, tcpI, and acfB, encrypting the establishment of the coregulated pilus toxin, with cholera toxin (CT) [117,118]. Recently, *V. parahaemolyticus* has been the major cause of up to 20–30% of food poisoning human outbreaks of bacterial origin in Japan, seafood stomach disease in Asia, and gastroenteritis from the consumption of seafood in the United States [119,120,121]. On rare occasions, *V. parahaemolyticus* is associated with ear or wound infections which, at times, pose threats to individuals that are immunosuppressed, immunocompromised, or have underlying medical conditions [122].

*V. parahaemolyticus* is also known to harbor different virulence factors such as TDH-related hemolysin (trh) and thermostable direct hemolysin (tdh), adhesins, and two other type III secretions systems, T3SS1 and T3SS2, with varying degree of pathogenicity [124]. They both display similar hemolytic activity in living cells and lead to the lysis of human erythrocytes, most especially in brackish medium [125]. Another known virulence factor found in *V. parahaemolyticus* is thermolabile haemolysin (TLH) and this is veiled by the TLH gene which is also potent in disrupting red blood cells [126,127]. Studies have shown that both environmental and clinical strains of *V. parahaemolyticus* are known to express TLH [128], and the gene is considerably coordinated under assumed intestinal infection settings [129]. In addition, *V. parahaemolyticus* possesses two separate kinds of flagella that are used for reeling, swarming, and producing capsules. These features are envisaged to ensure the survival of strains in the environment, as well as to thrive in the human host.

The mode of action used by *V. vulnificus* to establish its pathogenicity in a human is reliant on host vulnerability, and this bacterium is considered as an opportunistic pathogen [130]. Generally, *V. vulnificus* infection in humans arises as a result of consuming improperly cooked seafood or a wound infection from sea water or contaminated fish [130,131]. The invasive nature of *V. vulnificus* is attributed to its ability to harbor varying multiple virulence factors such as iron availability in the host, the capsular polysaccharide, and a short generation time [132].

Several studies have described the prominent virulence factor expressed by *V. fluvialis* as hemolysis, which manifested on sheep blood agar. Different acknowledged virulence factors of *V. fluvialis* are as follows: cell vacuolation, hemaglutination cell adherence [133,134], mannose sensitive [135], hemolysin [136], cytotonic [137], mucinase [138], heat-labile Cytotoxin [139,140], and cytolysin [141]. Reference [25] discovered that the ability possessed in expressing all the virulence factors by *V. fluvialis* is not uniform. Generally, *Vibrio* pathogens thermostable direct haemolysin and cholera toxin are used to define *V. parahaemolyticus* and *V. cholerae*, respectively, while within *V. vulnificus* strains, host proneness appears to be a crucial factor for virulence.

## 5. Epidemiological Features of *Vibrio* Species

*Vibrio* species comprise genetically and metabolically different collections of heterotrophic bacteria that grow naturally and are able to proliferate in marine ecosystems and freshwater with increased salinity [142]. Since 1817, up to seven major plagues and cholera outbreaks have been reported in Asia and Africa, with minor cases in Australia and America. Sub-Saharan Africa is broadly affected by many cholera epidemics [143], where the risk associated with cholera infection is high. The prevalence of *Vibrio* species in the aquatic environment has a direct correlation to the numerous physicochemical and biological features of the water ecosystem. *V. cholerae* is found in surface water in a potent state between one hour to 13 days, while the incessant pollution by healthy carriers and victims of cholera epidemics serves as the main means of sustaining its proliferation in aquatic environments for up to 15 months in receiving waterbodies [144]. Consequently *Vibrio* pathogen infections remain a significant health challenge in middle-income countries, notably in Africa and Asia, endangering the basic health of weak people in the society [145]. *Vibrio* species have been implicated in cases of bloody diarrhoea, necrotizing fasciitis, and primary septicemia in immunocompromised individuals, especially in developing countries with inadequate sanitation, socioeconomic conditions, and water supply systems [28]; however, this is responsible for the varying degree of ill health and death in all age groups worldwide [146]. Natural tragedies such as tsunami and floods also aid outbreaks by unsettling the normal balance of nature [147]. This results in varying health challenges, making food and water supplies prone to contamination by parasites and bacteria when vital systems like those for water and sewage are destroyed. An example of such is the current outbreak of cholera in Yemen that has claimed over 1500 lives with more than 246,000 new cases and this now affects 21 out of the 22 provinces in Yemen [148].

Developing countries are extremely affected because of their paucity of resources, infrastructure, and disaster awareness systems [149]. The spread of cholera within neighbouring countries has been attributed to cross-border practices which include the migration of fisherman and commercial trade. The outbreaks of cholera and associated deaths that occurred between 1994 and 2016 in ten selected countries in sub-Saharan Africa countries are listed in Table 4. This further establishes the vulnerability of the developing countries and most especially children. In 2014 alone, about 190, 549 cases, with 2231 deaths, were reported all over the world, though available modeling suggests that the cases of cholera outbreak may be far higher with almost 1–4 million occurring every year. The current outbreak of cholera in DR Congo is very disheartening, with reported cases of high fatality rates. This has subjected the DR Congo health system to intense pressure. Overall, the prevailing noticeable upsurge of the size of outbreaks can be partially described in part by the resistance developed by *Vibrio cholerae* O1 strains to ciprofloxacin and the different cholera toxin B (ctxBtt) genotype [150]. Recent studies carried out by [151] acknowledged that most environmental strains of *V. cholerae* recovered from the Apies river in South Africa haboured virulent-related genes (hlyA, ToxR, tcp, and zot). The prevalence of these strains in our environments presents hidden public health threats to rural dwellers of developing countries that have little or no access to safe water for household uses. In addition, the occurrence of the virulent-related genes in the absence of the ctx gene in isolated *V. cholerae* calls for further research in an attempt to unravel possible triggers of cholera epidemics in most developing countries where known toxigenic strains of the bacterium are not common.

Additionally, the recent findings that affirm the occurrence of *V. cholerae* in river sediments further confirm that the risk of infection associated through exposure to the river could increase under circumstances of sediment resuspension [153]. The possibility of isolating *V. cholerae* strains from river sediments may possibly increase the understanding of the possible sources of the *V. cholerae* strains involved in the cholera epidemics that have affected many developing and Sub-Saharan African countries for many years.

The absence of cholera enterotoxin was also reported in *V. cholerae* non O1/O139 isolated from the water of several reservoirs in Burkina Faso. The fact that the bacterium did not harbor the ctx gene does not exclude the threat associated with the presence of the *V. cholerae* non O1/O139 in environmental waters [154,155]. Some studies have earlier demonstrated the antigenic translation of *V. cholerae* non O1/O139 to *V. cholerae* O1 in favourable conditions [156,157,158].

Between 1976 and 1997, Bangladesh witnessed the most devastating outbreak of *V. fluvialis* [159]. Moreover, between 1997 and 2000, *Vibrio* observation statistics indicate that *V. fluvialis* was liable for 82 out of 1584 *Vibrio* infections in the report submitted to Centers for Disease Control and Prevention. In Kolkata in India, [160] reported the increase in the rate at which *V. fluvialis* is isolated from hospitalized patients with cholera-like symptoms. [161] revealed the survival of *V. fluvialis* in wastewater effluents in South Africa and there is a previous report linking this bacterium to causing food poisoning [162], especially due to the consumption of inadequately prepared shellfish [163]. Generally, recent studies have established the epidemiological relevance of *V. fluvialis* in several countries regardless of their economic circumstances [25,131].

Human infections caused by *V. vulnificus* occur virtually everywhere has and have been isolated from estuarine or coastal environments, which was first reported in the United States by the Centre for Disease Control in 1964. Though it was erroneously recognized as a virulent strain of *V. parahaemolyticus*, it was later understood in 1970. The disease conditions displayed include wound infections and septicemia, which were unique and different from other *Vibrio* species [164,165,166]. This bacterium is unique for a number of reasons, such as its exceptional pathogenicity, high case fatality rate, interesting and unusual epidemiology, hidden virulence potential, and the increasing incidence of disease. Indeed, this pathogen is strikingly interesting because recorded cases occur in males (~85%) and in patients with repressed illnesses resulting in raised serum iron levels, primarily hepatitis and alcohol-related liver cirrhosis. Oestrogen appears to reduce the ability of this pathogen to elicit endotoxic shock in women; however, the molecular basis of this protective role remains unclear. Besides foodborne disease, *V. vulnificus* causes potent fatal wound infections. Generally, *V. vulnificus* wound infections are categorized by swelling, erythema, and acute pain. Significantly, as compared to other vibrios, *V. vulnificus* needs only minute portals of entry to initiate wound infections, and often initially appears as an insect bite [167]. *V. vulnificus* is heterogeneous and the basic features—genetic, biochemical, serological, as well as host range—are used to categorize it into three biotypes. Biotypes 1 and 2 are known to cause infections in human and aquatic animals, respectively. However, the third biotype is a hybrid of biotype 1 and 2. This was first discovered in 1996 from *V. vulnificus* infections at a fish market in Israel [168]. Recent studies have further revealed the complication surrounding the virulence process of *V. vulnificus* as biotype 1 has been discovered to habour two distinct genotypes termed C (clinical) and E (environmental). In addition, the genotype strains C are mostly associated with human septicaemia, while the genotype E is encountered in wound infections caused by *V. vulnificus*. [28] revealed the occurrence of *V. vulnificus* in effluents from wastewater treatment plants even after chlorination in South Africa. Several discrete genes are believed to be important in pathogenesis, as well as those involved in cytotoxicity, haemolysins, iron sequestration pathways, secretion systems, and acid neutralization pathways. Up to date, there has been no record of any single molecular target that has been documented which is capable of differentiating pathogenic and non-pathogenic *V. vulnificus* strains and this calls for more research in an attempt to unravel the differences that exist within these strains.

*Vibrio parahaemolyticus*, a halophilic bacterium, has gained global brutality, having being linked to an emergence of gastroenteritis throughout the world, including Africa, Europe, and Asia [169]. Several studies have tried to establish that this human pathogen resides in different geographical locations. *Vibrio parahaemolyticus* was first isolated and recognized as the causative agent of seafood-borne infections in Japan in 1950, which accounted for over 272 illnesses and 20 deaths after the consumption of shirasu, a local delicacy [170]. From 1996 to 1998, the Infectious Disease Surveillance Centre in Japan declared *V. parahaemolyticus* as the main cause of food poisoning [171]. Lethal *V. parahaemolyticus* is spread via the consumption of partially, undercooked, or contaminated marine products, and is capable of initiating acute gastroenteritis [172,173]. The disease condition caused by *V. parahaemolyticus* is associated with three major clinical manifestations which include gastroenteritis, wound infections, and septicemia. The most prominent of these syndromes is gastroenteritis, with symptoms such as watery diarrhea (occasionally bloody diarrhoea) with abdominal pains, nausea, spewing, headaches, and fever [171,174]. The mean period of *V. parahaemolyticus* illness is 15 h (range: 4–96 h) [175]. *V. parahaemolyticus* infection in immunocompetent folks is self-limiting, mild, and of moderate severity, lasting an average of three days [176,177]. About 45,000 annual cases of food-borne disease associated with *V. parahaemolyticus* infections are reported in the United States, and this is a public health threat because the incidence keeps increasing in spite of control measures; this is attributed to the impact of climate change on pathogen abundance and distribution. Almost 20 to 30% of all reported food poisoning cases in Japan are caused by *V. parahaemolyticus* [178] and it is considered as one of the major causes of seafood and marine products-borne illness [179,180]. Furthermore, several cases of gastroenteritis which result from the consumption of contaminated seafood, as well as cholera outbreaks, were also reported in Nigeria [181].

## 6. Treatment and Antibiotic Resistance of *Vibrio* Species

The frequency of *Vibrio* species infections, which cause illnesses that vary from acute diarrhea, septicemia, and gastroenteritis to primary sepsis and necrotizing fasciitis, continues to increase particularly in developing and middle-income countries where infectious diseases and poverty are endemic [182]. The treatment of the cholera disease condition is centered on the physiological ideology of replacing water and electrolytes and maintaining the intravascular volume. The main goal is to replenish potassium and bicarbonate, which were discharged along with choleric stool. For severely ill patients, the Centre for Disease and Control (CDC) recommends the use of antibiotics along with fluid replacement. The application of this physiological principle is primarily made available to patients who are sternly dehydrated and who continue to discharge large volumes of stool throughout the rehydration treatment. The use of antibiotic treatment is also recommended for all patients who are hospitalized. Antimicrobial agents are useful in aiding the rehydration treatment of cholera, because their use reduces the duration of diarrhoea (which in turn reduces the spread of the disease), and treats acute illnesses (by reducing the volume of diarrhoea). CDC recommends that the class of antibiotics used for treating any infection should be based on indigenous antibiotic susceptibility patterns. The first line of treatment for adults in most countries as recommended is doxycycline; however, azithromycin is recommended as the primary treatment for pregnant women and children. For the period of an epidemic, an antibiogram must be observed by carrying out regular tests on all sample isolates from different geographic regions [73].

Ever since the discovery of antibiotics and other antimicrobial therapies, they have always been used to treat both old and new emerging infections, subsequently leading to disease management and control [183,184]. However, treating antibiotic resistant infections with existing antibiotics has become more challenging, giving the rise in infections that result in higher morbidity and mortality [185,186]. In sub-Saharan Africa, the prevalence of the high burden of infectious diseases, which are mainly of bacterial origin, has increased the demand for antimicrobial remedies for treatment [182]. Furthermore, in the healthcare environment, shortfalls ranging from the inadequate diagnostic capacity and resources, high out of pocket cost of antimicrobial drugs, and lack of free access to antibiotics, to constrained access to health services and poor orientation with respect to antibiotic use [187,188,189], have gradually fueled the demand for antibiotics. Every year, quite a large number of *Vibrio* species are documented to harbour high resistance genes towards commonly used antibiotics. Drug resistance is one of the most alarming public health concerns that advances rapidly and threatens the advancement of disease management and control [190,191].

The upward trend of antibiotics resistance by microbial pathogens portends to weaken the idealistic hope of public health gains made since the widespread use of antibiotics was adopted. The emergence of antibiotics resistance among various species of *Vibrio* pathogens is a well-established phenomenon [192] and with the ongoing challenges of producing potent and effective new antibiotics [193], the management of communicable diseases has become a dire need in less industrialized countries where poor sanitation and malnutrition are prevalent. The indiscriminate use of antibiotics and chemotherapeutic agents as feed additives or immersion baths to establish preventive measures in farming and aquaculture environments has also been implicated in the emergence of multidrug resistance in aquatic microorganisms such as the *Vibrio* species [194].

Recent studies have established the role played by municipal and industrial wastewater and aquaculture as drivers of resistance genes in the aquatic ecosystem. A sizeable portion of the clinically used antibiotics consumed by humans is released in an active biological form through urine and faeces [195,196,197]. The residual portion of the antibiotics excreted by humans is discharged into wastewater treatment plants, with one of three fates: (1) biodegradation [198], (2) absorption to sewage sludge [199,200], or (3) exit as inadequately treated effluent [201,202]. Furthermore, 16 selected United Kingdom wastewater treatment plants (WWTPs) showed the existence of erythromycin, ofloxacin, and oxytetracycline residues in each of the WWTPs [203].

Moreover, about 30–90% of antibiotics ingested by animals are found in their faeces and urine [204]. Animal excreta are also known to pollute the environment with antibiotic resistant bacteria and antibiotics [205,206]. This phenomenon was recently verified in a study conducted in the Netherlands using 20 salable swine and 20 calf farms. The result revealed antibiotics in 55% of the swine faeces out of the 80% of the swine farms considered, and in 75% of the calf faeces from 95% of the cattle farms [204]. Among the antibiotics residues recovered, oxytetracycline, doxycycline, and sulfadiazine remained the most recurrent. This is in line with some noteworthy reports affirming that the use of antibiotics and biocides in fish farms tends to increase the circulation, which in turn contributes to the wide range of resistance genes within our environment.

Antibiotic resistance is known as the enhanced ability of an organism to withstand the effect of antibiotics to which it was previously vulnerable. In the treatment of different Vibrios infections, antibiotics such as Amoxicillin, Ampicillin, Chloramphenicol, Cotrimoxazole, Ciprofloxacin, Doxycycline, Erythromycin, Fluoroquinolone, Furazolidone Gentamicin, Kanamycin, Nalidixic acid, Neomycin, Norfloxacin, Polymyxin B, Quinolone, Streptomycin, Spectinomycin, sulfamethoxazole–trimethoprim, Sulphonamides, Tetracycline, Trimethoprim, and Vancomycin are generally drugs of choice [73,207,208,209]. Several reports have established that both clinical and environmental *Vibrio* strains harbor antibiotic resistance genes [210,211,212]. The presence of this bacterium in the aquatic environment increases human fright on food safety owing to the latter possibly causing disease epidemics depending on the environmental conditions [213]. The advent of antibiotics resistance is a challenging process repeatedly linking human, environmental, and pathogen-related features [184,187,192]. In general, the antibiotic routine in humans and animals conveys an intrinsic threat of opting for antimicrobial resistance genes (ARGs). The predominance of resistance genes in the environment is the outcome of an intricate combination of dynamics, which reveals an active balance of fitness costs and aids: costs of transporting the ARGs in the framework of the host genome and environment [214,215], relative to the sternness and recurrence of risk [216], pertinent to some physical environmental features, such as temperature [217] and microbial ecology [218], among others.

## 7. Mechanism of Antibiotics Resistance in *Vibrio* Species Infection

Several antibiotic resistance mechanisms in bacteria are usually enabled by exporting drugs through efflux pumps, chromosomal mutations, or developing genetic resistance via the exchange of conjugative plasmids, conjugative transposons, integrons, or self-transmissible chromosomally integrating SXT elements [209,219]. Some antibiotic-resistant *Vibrio* species in sub-Saharan Africa, as well as their resistance mechanisms, are listed in Table 5. *Vibrio* species are known to employ multi-drug efflux pumps to establish resistance against antimicrobial agents and other toxic compounds by a mechanism that prevents the accumulation of drugs inside the bacterial cells. *V. cholerae* has shown its ability in using multidrug efflux pumps to export a wide range of antibiotics, detergents, and dyes that are chemically and structurally unrelated [220]. Collectively, multi-drug efflux pumps are not employed only for drug resistance, but have also been implicated in the expression of important virulence genes in *Vibrio* pathogens. The spread of antibiotic-resistant pathogens in *V. cholerae* is known to be facilitated by horizontal gene transfer through self-transmissible mobile genetic elements, including SXT elements—mobile DNA elements belonging to the class of integrative conjugating elements (ICEs). The SXT genetic mobile element ICE conferring resistance to sulfamethoxazole-trimethoprim was first documented in *V. cholerae* O139 or a closely related ICE in Madras, India, owing to its ability to harbor resistance to trimethoprim, sulfamethoxazole, and streptomycin. The relationship between self-transmissible elements and multidrug resistance has been well documented in *Vibrio* species [221]. A recent study in Cameroun revealed that *Vibrio cholerae* O1 of environmental origin harbours heterogeneous multidrug resistance towards Amoxicillin (AML), Ampicillin (AMP), Tetracycline (TE), Chloramphenicol (C), Doxycycline (DXT), and Cotrimoxazole (SXT) [222]. The frequent usage of antibiotics as part of the *Vibrio* infection treatment regimen has resulted in the development of multidrug resistance in *V. cholerae* and seafood pathogens such as pathogenic *Vibrio* species [194].

As an environmental organism, *V. cholerae* has the means to acquire resistance genes from intimate contact with indigenous resistant environmental bacteria [235] through mobilizable genetic elements. The persistent discharge of antibiotics into WWTPs is associated with the release of resistance genes. These resistance genes in wastewater primarily originate from the gastrointestinal tracts of humans [236,237,238]. However, most of the genetic determinants that confer resistance to antibiotics are located on plasmids. Acquired antibiotic resistance in bacteria is generally mediated by extrachromosomal plasmids and is transferable to other bacteria within the environment [239]. The co-location of antibiotics and ARGs in WWTPs can select for novel combinations of AMR that can be shared between microorganisms by horizontal gene transfer (HGT) on mobile genetic elements (MGEs), such as plasmids, thereby increasing the prevalence and combination of multiple drug resistance in the microbial community [240,241]. Plasmid-mediated multidrug resistance is one of the most pressing problems in the treatment of infectious diseases. In the last decade, the emergence of antibiotic resistant genes in *Vibrio* species has been on the increase compared to previous years, and these genes include penicillin resistant genes penA, blaTEM-1 and Beta-lactam [242,243], chloramphenicol resistant genes [244], and tetracycline resistant genes [245]. There has been little or no regulation of the choice of antibiotics administered to animals, with overlaps in the classes of antibiotics used for farming and human therapy in most of the sub-Saharan countries. The risk of multiple drug resistance found in environmental microorganisms being transferred to other pathogens is of significant public health concern that calls for concerted efforts in tackling the threat posed to disease control and management [246,247,248]. These animals, animal products, farm workers, and the farming environment itself are potential reservoirs for resistance determinants. Antimicrobial resistance has been detected in farms; however, the extent of resistance and spill over in the country remains largely unknown. Hence, the transmission of resistance between animal feed and humans is important and requires investigation, as this has been linked to increasing clinical resistance in human medicine.

## 8. Strategic Recommendations for High-Risk Cholera Outbreak Areas

High-risk cholera areas along the coastline are regions that serve as pathways for exchange with neighbouring countries. The neighbouring areas that are prone to cholera outbreaks with high incidences call for cross-border collaboration needs for preparedness and early detection [249]. The priority strategic actions to be taken in the above onset regions include: (i) consolidating timely uncovering and prompt response schemes with community-based surveillance and cross-border alerts; (ii) establishing coordination machineries through the sectors and borders; (iii) building the capacity for outbreak management; (iv) targeted the pre-positioning of supplies; and (v) preparing communication messages and plans. Sustainable Water, Sanitation, and Hygiene activities should be of main concern in rural areas that are often affected by long outbreaks. An integrated WASH-epidemiological study has been steered by UNICEF in the West Africa region and proposes to (i) institute support with concerned Water Company Limited to advance the water quantity and quality delivered to the rural areas; (ii) reinforce post-chlorination of the network by mounting dosing chlorine pumps at premeditated points along the network; (iii) advocate the use of GIS technology during an outbreak to ascertain any strategic hotspots; and (iv) when hotspots are known, implement WASH and Health development programs targeting identified communities, and consider the use of Oral Cholera Vaccine [250].

## 9. Conclusions

The concerns about public health risks from *Vibrio* pathogens, most especially when rural waters, wastewater effluents, and mishandled sea products remain the means of transmission of *Vibrio* species infections, are expected to remain in the future. Every year, there has been an emergence of at least one new pathogenic *Vibrio* species which could be transferred through the environment as a new public health menace. This is as a result of a number of factors which include: (i) the impact of global climate change on environmental conditions; (ii) the indiscriminate use of chemotherapeutic agents and antibiotics in aquaculture environments and agriculture; (iii) the enhanced ability of the *Vibrio* species to transfer acquired resistance genes; (iv) the evolution of pathogens; and (v) the application of a global effective surveillance system to ascertain the risks of environmentally transmitted pathogenic *Vibrio* species and the indiscriminate use of antibiotics for treatment and prophylaxis measures. The observed transmission of antibiotic-resistant bacteria and genes from animals to humans highlights the importance of biosecurity and the need to separate animals being treated for infection from the herd (where feasible), and not to re-use beddings from infected and treated animals. The use of an effective global surveillance system to monitor factors such as ecological modifications and climate change that enhance *Vibrio* species as significant human pathogens demands quantitative assessments rather than assumptions. There is a dire need of information on the indiscriminate traditional use of antibiotics in farming and aquaculture environments as immersion baths or feed additives, which is believed to contribute to the prevalence of antibiotic resistance in humans within sub-Saharan Africa. Furthermore, to reduce the proliferation and survival of emerging and currently recognized pathogens in the final effluent being discharged into receiving water bodies, there is an urgent need to adopt advanced treatment methods and maintenance strategies. The implementation of adequate surveillance management protocols to reduce the possibility of infections caused by pathogenic *Vibrio* species is also proposed. Finally, the use of advanced molecular techniques and the integration of skills from related fields (e.g., microbiology, biotechnology, and ecology) will promote a better understanding of the state and possible causes of pollution which can, in turn, help in developing long-term policies to improve water quality.

## Figures and Tables

**Table 1:** Some disease conditions initiated by pathogenic *Vibrio* species.

***Vibrio* Species**	**Intestinal Syndromes**		**Extra-Intestinal Syndromes**		
	Diarrhea	Cholera	Septicemia	Skin-infection	Others *
*Vibrio cholerae* O1/139	-	##	-	-	≠
*Vibrio cholerae* non O1/non 139	##		#	#	≠
*Vibrio alginolyticus*	-		≠	##	≠
*Vibrio damsela*	-		##	##	-
*Vibrio fluvialis*	##		-	-	-
*Vibrio metschnikovi*	≠		-	-	≠
*Vibrio mimicus*	##		-	-	≠
*Vibrio parahaemolyticus*	##		##	##	≠
*Vibrio vuinificus*	≠		##	##	≠

**##** key infections; **#** minor infections; ≠ random infections. * includes otitis media cholesystitis, meningitis. Adapted from [49].

**Table 2:** Biochemical characterization of some *Vibrio* species.

Caption	*V. cholerae*	*V. parahaemolyticus*	*V. fluvialis*	*V. furnisii*	*V. vulnificus*	*V. alginolyticus*	*V. cincinnatiensis*	*V. damsela*	*V. hollisae*	*V. metchnikovii*	*V. mimicus*
TCBC agar	YLW	GRN	YLW	YLW	GRE	YLW	YLW	GRE	ABS	GRE	GRE
mCPC agar	Purple	ABS	ABS	ABS	YLW	ABS	NOTD	ABS	ABS	ABS	ABS
AGS	AKa	AKa	AKAK	AKAK	AKa	AKa	NOTD	NAD	Aka	AKAK	AKa
Grth. NaCl (0%)	+	−	−	−	−	−	−	−	−	−	+
Grth. NaCl (3%)	+	+	+	+	+	+	+	+	+	+	+
Grth .NaCl (6%)	−	+	+	+	+	+	+	V	+	+	−
Grth NaCl (8%)	−	+	V	+	−	+	−	−	−	V	−
Grth. NaCl (10%)	−	−	−	−	−	+	−	−	−	−	−
Grth at 42 °C	+	+	V	-	+	+	−	−	NOTD	V	+
CA	NOTD	+	−	NOTD	+	−	NOTD	NOTD	NOTD	NOTD	NOTD
VP	NOTD	-	-	NOTD	-	+	NOTD	NOTD	NOTD	NOTD	NOTD
SU	+	−	+	+	−	+	+	−	−	+	−
CE	−	V	+	−	+	−	+	+	−	−	−
LA	−	−	−	−	−	−	−	−	−	−	−
AB	−	−	+	+		−	+	−	+	−	−
MA	+	+	+	+	+	+	+	+	+	+	+
MA	+	+	+	+	Va	+	−	−	+	+	+
OX	+	+	+	+	+	+	+	+	+	−	+
Ad	−	−	+	+	−	−	-	+		+	−
Ld	+	+	−	−	+	+	+	−	−	+	+
Od	+	+	−	−	+	+	−	−	−	−	+
S 0/129 (10 μg)	SNTIVE	REST	REST	REST	SNTIVE	REST	SNTIVE	SNTIVE	NAD	SNTIVE	SNTIVE
S 0/129 150 μg)	SNTIVE	SNTIVE	SNTIVE	SNTIVE	SNTIVE	SNTIVE	SNTIVE	SNTIVE	NAD	SNTIVE	SNTIVE
GE	+	+	+	+	+	+	−	+	−	+	+
UR	−	V	−	−	−	−	−	+	−	−	−

Note: AGS—arginine-glucose slant; Grth—Growth; CA—Capsule; VP—Voges-Proskauer; SU—Sucrose; CE—Cellobiose; LA—Lactose; AB—Arabinose; MA—Mannitol; OX—Oxidase; Ad—Arginine dihydrolase; Ld—Lysine decarboxylase; Od—Ornithine decarboxylase; GE—Gelatinase; UR—Urease; AC—Acid; AK—Alkaline; Va—Variable reaction; a—slight acid; ABS—Absence of growth; GRE—green; YEL—yellow; NOTD—Not determined; SNTIVE—Sensitive; REST—Resistant. Adapted from [40,41].

**Table 3:** Some proteolytic enzymes produced by pathogenic *Vibrio* species. Adapted from [49,123].

*Vibrio* Species	Vibrolysin	Collagenase	Chymotrypsin-Like Protease	Haemolysin
*Vibrio alginolyticus*		Present	Present	
*Vibrio parahaemolyticus*		Present	Present	Present
*Vibrio mimicus*	Present			
*Vibrio cholerae*	Present			Present
*Vibrio vulnificus*	Present		Present	
*Vibrio fluvialis*	Present			Present
*Vibrio metschnikovii*			Present	
*Vibrio anguillarium*				Present
*Vibrio tubiashii*				Present

**Table 4:** Epidemiological updates of cholera outbreaks from 10 selected countries in Sub-Saharan Countries between 1994 and 2016. Adapted from [152].

Name of Countries	Cases between (1994–2013)				Cases in 2014			Cases in 2015			Cases in 2016		
	Year	Cases	Death	CFR	Cases	Death	CFR	Cases	Death	CFR	Cases	Death	CFR
Nigeria	2004–2013	105,483	3913	3.7	35,996	755	02	5913	188	3.2	768	32	4.2
Cameroun	2004–2013	46,172	1817	3.9	3355	184	05	120	5	4.2	77	1	1.2
Niger	1994–3013	21,538	978	4.5	2059	80	04	51	4	7.8	38	5	13.2
Lake Chad Basin	2004–2013	31,918	996	3.2	41,188	994	2.4	6084	197	3.2	883	38	4.3
Ghana	1998–2013	55,784	1095	2	28,944	247	01	687	10	1.5	600	00	00
Benin	2004–2013	5432	48	0.9	874	14	02	00	00	00	874	13	1.5
Togo	2006–2013	2142	38	1.8	329	11	03	50	02	4.0	02	00	00
Cote d’Ivoire	2002–2013	7573	272	3.6	248	14	06	200	02	1.0	16	01	06
Guinea Bissau	1996–2013	74,031	1684	2.3	18	3	7	00	00	00	00	00	00
DR Congo	-	-	-	-	19,305	265	01	18,403	272	1.5	28,162	772	2.7

Note: The risk factors for cholera outbreaks in the 10 selected countries in sub-Saharan Countries between 1994 and 2016 are poor sanitation, lack of safe water and cross border.

**Table 5:** Selected drug-resistant *Vibrio* species strains reported in Sub-Saharan Africa. Adapted from [209] with slight modification.

Year	Country	Strain	Antibiotic Resistance	Mechanism	Reference
2006	Accra, Ghana	O1	SXT	SXT element, Class 2 integron, Class 1 integron.	[223]
2011–2014	Ghana	O1 biotype El Tor	Am, Cpr, NA, SXT.	ND	[224]
Nov. 2002–April 2004	Mozambique	O1 El Tor Ogawa	Cm, Co, Tet, Qu.	ND	[225]
1994	Rwanda	O1 EL Tor	Co	ND	[226]
Oct. 2004–Mar. 2006	Senegal	O1 El Tor	Co	ND	[227]
2009–2010	Nigeria	atypical El Tor	SXT, Spec	ND	[154]
2004–2005	Cameroun	O1	SXT, Amp	ND	[228]
Aug. 2006–Sep. 2008	North-west Ethiopia	O1 Inaba	Co, Cm, Amp, Ery, Tet, Cpr.	ND	[229]
2011–2012	DR Congo	Ogawa and Inaba	NA, Am, Cm, Tet, Do, Nf, SXT, Ery.	ND	[230]
Dec. 2006–Feb. 2007	Namibia	O1 El Tor Inaba	SXT, Sm.	ND	[231]
1998–1999	Kenya	O1	Spec, Cm, Co, Tet	ND	[232]
2006	Angola	O1 and *Vibrio parahaemolyticus*	Am, Cm, Tri, SXT, Tet.	Plasmid located Class 1 integrons.	[233]
2010	South Africa	*Vibrio fluvialis, Vibrio* species	Vf (Tri, Pen, Co, Spec). V spp. (Am and SXT).	ND	[28]
2008–2009	South Africa	O1 Inaba	Co, NA, Am, Tet, Cm, Ery, Ce.	Tet A gene, SXT element-integrase	[234]

Note: Am: amoxicillin; Amp: ampicillin; Cm: chloramphenicol; Co: cotrimoxazole; Cpr: ciprofloxacin; Ery: erythromycin; Spec: spectinomycin; SXT: sulfamethoxazole–trimethoprim; Tet: tetracycline; Tri: trimethoprim; NA: nalidixic acid; Qu: quinolone; Sm: streptomycin; pen: penicillin; Ce: cephalosporin.

## Abbreviations:

**VBNC**	Viable but non-culturable
**TCP**	Toxin coregulated pili
**CT**	Cholera toxin
**CTX**	Cholera causing toxin phage
**LGT**	Lateral gene transfer
**DNA**	Deoxyribonucleic acid
**TDH**	Thermostable direct haemolysin
**TLH**	Thermolabile haemolysin
**CDC**	Centres for Disease control
**WWTPs**	Wastewater treatment plants
**ARGs**	Antibiotic resistance genes
**SXT**	Self-transmissible chromosomally integrating SXT elements
**ICE**	Integrative conjugating elements
**AMR**	Antimicrobial resistance
**HGT**	Horizontal gene transfer
**MGEs**	Mobile genetic elements
**GIS**	Geographical information system
**WASH**	Water Sanitation and hygiene
**UNICEF**	The United Nations Children’s fund
